# Case Report: Acute obstructive hydrocephalus associated with infratentorial extra-axial fluid collection following foramen magnum decompression and durotomy for Chiari malformation type I

**DOI:** 10.12688/f1000research.7627.1

**Published:** 2016-01-07

**Authors:** Sunil Munakomi, Binod Bhattarai, Pramod Chaudhary

**Affiliations:** 1 Department of Neurosurgery, College of Medical Sciences, Bharatpur, 44207, Nepal

**Keywords:** Foramen Magnum Decompression, Chiari Malformation, Extra Axial Fluid Collection, Ventriculo-Peritoneal Shunt

## Abstract

Acute obstructive hydrocephalus due to infratentorial extra-axial fluid collection (EAFC) is an extremely rare complication of foramen magnum decompression (FMD) and durotomy for Chiari malformation type I. Presence of infratentorial  EAFC invariably causes obstruction at the level of the fourth ventricle or aqueduct of Silvius, thereby indicating its definitive role in hydrocephalus. Pathogenesis of EAFC is said to be a local arachnoid tear as a result of durotomy, as this complication is not described in FMD without durotomy. Controversy exists in management. Usually EAFC is said to resolve with conservative management; so hydrocephalus doesn’t require treatment. However, in this case EAFC was progressive and ventriculo-peritoneal shunting (VPS) was needed for managing progressive and symptomatic hydrocephalus.

## Introduction

Chiari malformation type I is not associated with other congenital conditions. Typically it presents in adulthood with varying features of increased intracranial pressure, headache, progressive cerebellar ataxia, progressive spastic quadriparesis, segmental amyotrophy and sensory loss, with or without pain. The latest management protocol is foramen magnum decompression (FMD) with or without durotomy. Several complications such as cerebrospinal fluid (CSF) fistula, cerebellar hematoma, and worsening neurological status have been described, but acute obstructive hydrocephalus due to infratentorial subdural hygroma is very rare ^1, 3– 6^. Herein we discuss one such case and review the literature regarding the current knowledge of such a rare complication in the posterior fossa.

## Case report

**Figure 1.**
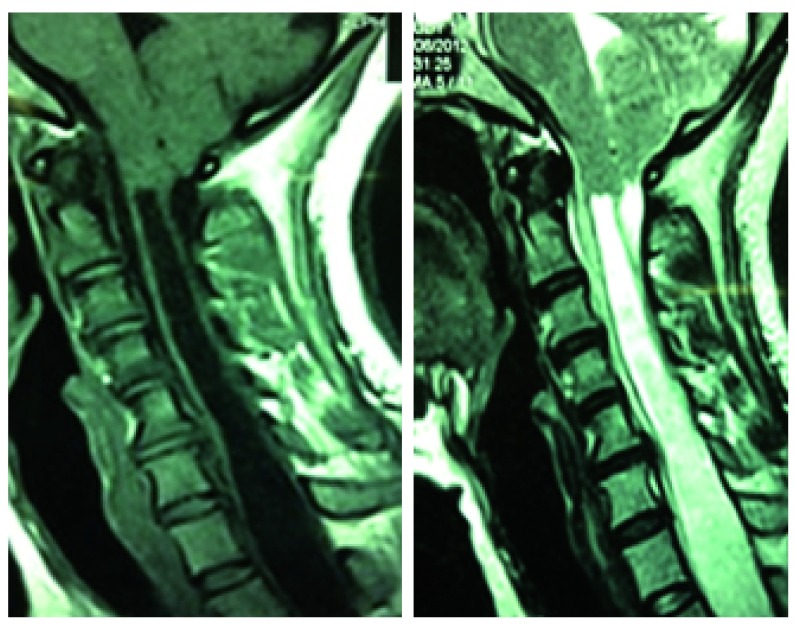
T1 and T2 MRI showing descended cerebellar tonsils and wide syrinx starting from C2.

**Figure 2.**
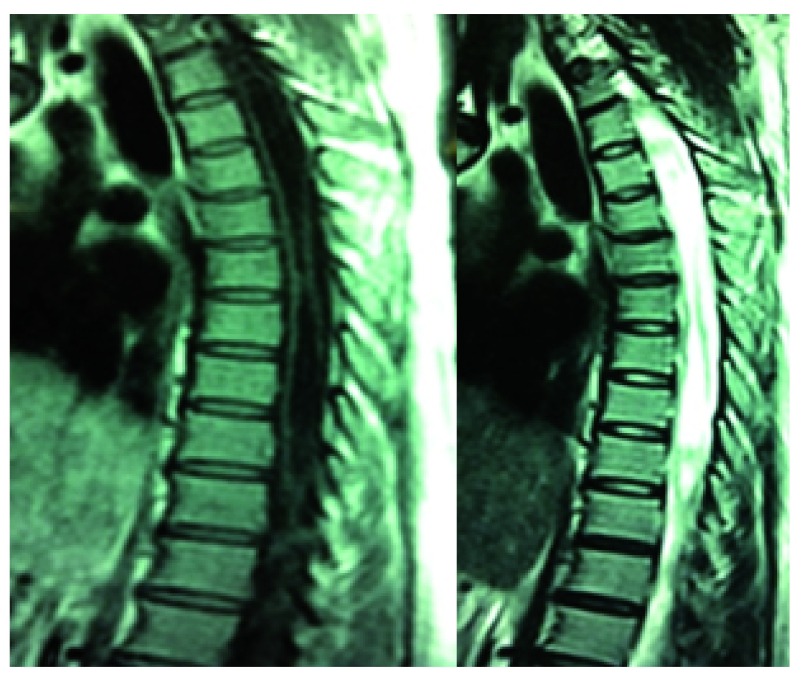
T1 and T2 MRI shows extension of syrinx up to lower T8 level.

A 30-year-old Tharu lady from Birgunj, Nepal was referred to the outpatient clinic at the College of Medical Science (Nepal) with symptoms of chronic dull neck pain without radiation for the previous two years. There was no history of trauma. There was no significant past medical or surgical illnesses. The patient was taking over the counter pain medications (Diclofenac 75 mg PO SOS) for the pain. Due to further aggravation of her symptoms, she opted for medical review. On general examination, she had short neck (distance between external occipital protuberance and C7 spinous process was only 60 mm) with low hair line. There was cape-like dissociative sensory loss starting from C2-T4 level. Muscle tone was of Ashworth grade I. She had exaggerated deep tendon reflexes in upper and lower limbs without appreciable clonus. Magnetic resonance imaging (MRI) of the cervical and thoracic spine was suggestive of Chiari malformation type I with syringohydromyelia extending from C2 to T8 level ( Figure 1 and Figure 2). There was no evidence of hydrocephalus. The patient was thoroughly counselled of her medical condition and surgical intervention was advised.

FMD and durotomy were performed after receiving patient consent. Improvement in spasticity was seen immediately from the 1 ^st^ postoperative day. The patient’s hospital stay was uneventful and she was discharged after suture removal on the 7 ^th^ postoperative day.

**Figure 3.**
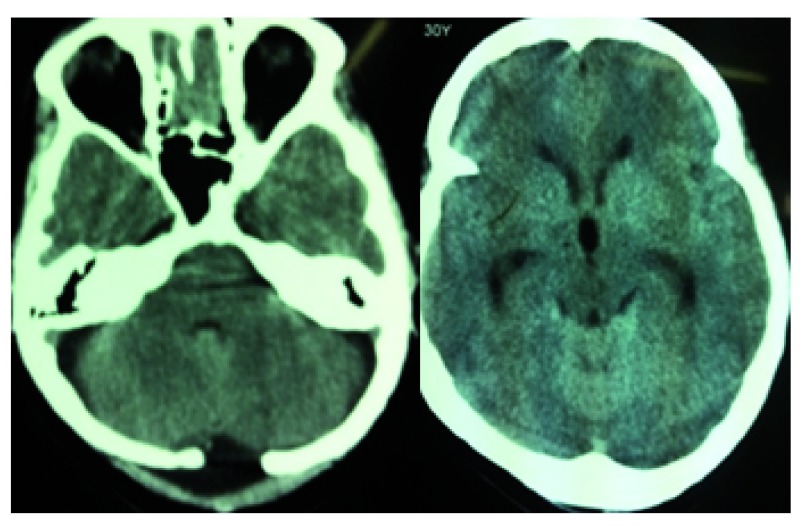
Infratentorial EAFC and evolving triventricular hydrocephalus.

Three weeks following surgery, the patient returned with complaints of headache and dizziness. She had no added neurological deficits and fundus examination was normal. A CT head scan showed minimal infratentorial EAFC and rounded third ventricle and prominent temporal horn. Initially she was managed conservatively with tablet acetazolamide 250 mg PO every 8 hours and strict monitoring for features of raised intracranial pressure such as persistent vomiting, hypertension and bradycardia. On the 6 ^th^ day of her admission, she deteriorated with severe headache and persistent vomiting Early papilloedema was evident on fundoscopy and a repeat CT scan showed an increase in EAFC and triventricular hydrocephalus ( Figure 3). Ventriculo-peritoneal shunting (VPS) was done for the same. Ventricular tapping revealed high pressure clear CSF. Her symptoms subsequently disappeared and she was discharged on the 7 ^th^ day. She continues to follow up in the outpatient department every 6 months with no new symptoms and better resolution of her previous ailments. She is able to differentiate the sense of temperature in the areas where she had dissociative sensory loss prior to the management. Her exaggerated deep tendon reflexes have gradually resolved over 2 months of her surgery. Her VPS is functioning well to-date (2 years after surgery).

## Discussion

FMD with or without durotomy is the most widely used surgical technique for Chiari malformation type I ^1^. Some surgeons prefer to perform a wide midline suboccipital craniectomy and not to open the dura with a view that duramater can slowly stretch so as to accommodate posterior fossa structures. Although some authors advocate performing a simple lax duraplasty alone, others provide a rationale for performing arachnoidolysis subsequent to duraplasty ^2^.

With our improved insights on the pathogenesis of Chiari malformation, our surgical nuances and techniques on the management of the condition have improved significantly. Complication rates are reported to be as low as 2.3% ^1^. Among these complications subdural hygroma or EAFC causing acute obstructive hydrocephalus is very rare ^1, 3– 6^. The exact cause of subdural hygroma is still not clear. It is believed that during durotomy or arachnoidolysis, a small hole may be created on the arachnoid which will cause CSF egress in the subdural space. This tear acts like a one way valve causing more CSF to collect in the subdural space. Initially, CSF accumulates in and around the foramen magnum. With pressure created, CSF will slowly flow towards the cisterna magna, bilateral cerebellar convexity, below tentorium cerebelli and finally through the tentorial hiatus to the supratentorial subdural space ^3^. Some authors have advocated the probable role of increased permeability of the intracranial vessels, which seems to be logical in cases of traumatic brain injury only ^7^. Subdural hygroma under tension causes compression at the level of the fourth ventricle or aqueduct of Silvius, thus causing acute hydrocephalus ^5^.

As seen from the literature review, this complication is usually seen in patients above 10 years of age and has special preponderance to females. No clear hypothesis or mechanisms has been proposed for this, however a tear or breach in the dura seems to be the most likely. Age varied from 10 years to 55 years. Until now, including our study, nine cases have been reported, of which seven describe female patients.

The site of maximal collection of extra-axial fluid collections differ between case reports. Such extra-axial collections may only be confined to infratentorial area, may extend to supratentorial area or only be prominent in supratentorial areas. Infratentorial extra-axial fluid collections usually cause compression at the level of fourth ventricle or aqueduct of Silvius and cause triventricular hydrocephalus. It may also result in direct compression of the brain stem and cranial nerves, causing acute symptoms. Supratentorial subdural effusion (EAFC) acts like decompensated chronic subdural hematoma thus causing relevant symptoms such as hemiparesis or signs of raised intracranial pressure.

EAFC may resolve spontaneously or aggravate causing acute hydrocephalus. Acute hydrocephalus may be transient as EAFC might slowly resolve. Patients usually are symptomatic requiring hospital admission from 3–21 days after FMD.

This is a rare complication so the ideal management protocol is not known. Effort should be made to prevent it by avoiding durotomy and arachnoidolysis whenever possible and opening the arachnoid widely whenever deemed necessary. Some authors suggest re-surgery for closing the tiny hole in the arachnoid or opening the arachnoid widely and suturing it to the dura ^4, 9, 10^. Widely opening the arachnoid routinely during surgery might prevent this complication as pinhole dural tear may even occur during closure ^3^.

After complication has already occurred, and is not life threatening, minimal measures such as burr hole and external ventricular drain (EVD) should be undertaken. In most cases reported till date, permanent VPS is not usually required as EAFC usually resolves ^6, 8, 10^. However, if there is evidence of significant mass effect and raised intracranial pressure, such patients should be managed surgically either with placement of appropriate burr holes or by performing VP shunting for CSF diversion. VPS should be considered for progressive neurological deterioration and increasing ventricular size. A subduroperitoneal shunt should be employed for recurrent supratentorial EAFC. There is inadvertent risk of slit ventricular syndrome due to over-drainage of CSF following low pressure VP shunting. Repeated EVD (more than twice) increases the risk of infection as well as the odds of upward central herniation in case of CSF over-drainage. So, VPS should be considered in such cases.

## Conclusions

EAFC complicating acute obstructive hydrocephalus is a very rare complication following FMD and durotomy for Chiari malformation type I; but should be suspected in post operative cases presenting with headache and vomiting. We can avoid this by only undertaking osseous decompression or in cases where there is need for durotomy, widely opening the arachnoid and suturing it to the duramater. Whenever applicable, attempts should be made to manage these cases without VPS.

## Consent

Both written and verbal informed consent for publication of images and clinical data related to this case was sought and obtained from the patient.

## References

[1] Tubbs RS McGirt MJ Oakes WJ: Surgical experience in 130 pediatric patients with Chiari I malformations. *J Neurosurg.* 2003;99(2):291–6. 10.3171/jns.2003.99.2.0291 12924703

[2] Siasios J Kapsalaki EZ Fountas KN: Surgical management of patients with Chiari I malformation. *Int J Pediatr.* 2012;2012:640127. 10.1155/2012/640127 22811732 PMC3395248

[3] Bahuleyan B Menon G Hariharan E: Symptomatic posterior fossa and supratentorial subdural hygromas as a rare complication following foramen magnum decompression for Chiari malformation Type I. *J Neurosurg.* 2011;114(2):510–3. 10.3171/2010.8.JNS10413 20849216

[4] Elton S Tubbs RS Wellons JC 3rd: Acute hydrocephalus following a Chiari I decompression. *Pediatr Neurosurg.* 2002;36(2):101–4. 10.1159/000048361 11893893

[5] Kabir SM Jennings SJ Makris D: Posterior fossa subdural hygroma with supratentorial chronic subdural haematoma. *Br J Neurosurg.* 2004;18(3):297–300. 10.1080/02688690410001732797 15327237

[6] Marshman LA Benjamin JC Chawda SJ: Acute obstructive hydrocephalus associated with infratentorial subdural hygromas complicating Chiari malformation Type I decompression. Report of two cases and literature review. *J Neurosurg.* 2005;103(4):752–5. 10.3171/jns.2005.103.4.0752 16266060

[7] Nishizaki T Tamaki N Fujiwara H: Posterior fossa subdural effusion due to head trauma. *Neurosurgery.* 1988;23(1):81–4. 10.1227/00006123-198807000-00013 3173666

[8] Perrini P Rawlinson A Cowie RA: Acute external hydrocephalus complicating craniocervical decompression for syringomyelia-Chiari I complex: case report and review of the literature. *Neurosurg Rev.* 2008;31(3):331–5. 10.1007/s10143-008-0129-5 18311492

[9] Zakaria R Kandasamy J Khan Y: Raised intracranial pressure and hydrocephalus following hindbrain decompression for Chiari I malformation: a case series and review of the literature. *Br J Neurosurg.* 2012;26(4):476–81. 10.3109/02688697.2011.650738 22280544

[10] Ranjan A Cast IP: Symptomatic subdural hygroma as a complication of foramen magnum decompression for hindbrain herniation (Arnold-Chiari deformity). *Br J Neurosurg.* 1996;10(3):301–3. 10.1080/02688699650040188 8799543

