# Supplementary material for: Case Report: Acute obstructive hydrocephalus associated with infratentorial extra-axial fluid collection following foramen magnum decompression and durotomy for Chiari malformation type I
Source: F1000Res. 2016 Jan 7;5:33. [Version 1] doi: 10.12688/f1000research.7627.1 (PMC4882752; doi:10.12688/f1000research.7627.1)
Supplement: Peer review history

# Peer review: Referee response for version 1

**DOI:** 10.5256/f1000research.8213.r13971

**Published:** 2016-05-25

**Authors:** Murat Şakir Ekşi

**Affiliations:** 1 Department of Orthopedic Surgery, Spine Center, University of California, San Francisco, San Francisco, CA, USA

The case report is informative about obstructive hydrocephalus development secondary to extradural fluid collection in the posterior fossa following decompression surgery for Chiari malformation type I. The authors mentioned about presence of scarce amount of cases in the literature. It would be better to delineate the prior cases in a table to give more information about the nature of the disease.

I have read this submission. I believe that I have an appropriate level of expertise to confirm that it is of an acceptable scientific standard.

# Peer review: Referee response for version 1

**DOI:** 10.5256/f1000research.8213.r12295

**Published:** 2016-02-29

**Authors:** Lekhjung Thapa

**Affiliations:** 1 Department of Neurology, National Institute of Neurological and Allied Sciences, Kathmandu, Nepal

The authors attempted to demonstrate a rare manifestation of operative intervention on adult type Chiari malformation. This kind of case report would undoubtedly help surgeons to understand the current status of managing this, which is still controversial. They have highlighted the importance of post operative headache and vomiting to identify a rare yet a treatable cause like EAFC. 

Comments:

1. Please mention how you defined the low hair-line.
2. It would be better for authors to mention why they chose ventriculoperitoneal shunting and not external ventricular drain to deal with the complication, as we know they may resolve spontaneously. (Marshman *et al. *2005 ^1^)
3. As the literature review seems less, I would rather say "Herein we report a case of Chiari malformation type I with postoperative infratentorial subdural hygroma"

I have read this submission. I believe that I have an appropriate level of expertise to confirm that it is of an acceptable scientific standard.

## References

[1] MarshmanLABenjaminJCChawdaSJDavidKM: Acute obstructive hydrocephalus associated with infratentorial subdural hygromas complicating Chiari malformation Type I decompression. Report of two cases and literature review. *J Neurosurg*.2005;103(4) : 10.3171/jns.2005.103.4.0752 752-5 10.3171/jns.2005.103.4.0752 16266060
